# Factors affecting the performance of brain arteriovenous malformation rupture prediction models

**DOI:** 10.1186/s12911-021-01511-z

**Published:** 2021-05-03

**Authors:** Wengui Tao, Langchao Yan, Ming Zeng, Fenghua Chen

**Affiliations:** Department of Neurosurgery, Xiangya Hospital, Central South University, Changsha, China

**Keywords:** Brain arteriovenous malformation, Logistic regression, Random forest, Prediction model, AUC

## Abstract

**Background:**

In many cases, both the rupture rate of cerebral arteriovenous malformation (bAVM) in patients and the risk of endovascular or surgical treatment (when radiosurgery is not appropriate) are not low, it is important to assess the risk of rupture more cautiously before treatment. Based on the current high-risk predictors and clinical data, different sample sizes, sampling times and algorithms were used to build prediction models for the risk of hemorrhage in bAVM, and the accuracy and stability of the models were investigated. Our purpose was to remind researchers that there may be some pitfalls in developing similar prediction models.

**Methods:**

The clinical data of 353 patients with bAVMs were collected. During the creation of prediction models for bAVM rupture, we changed the ratio of the training dataset to the test dataset, increased the number of sampling times, and built models for predicting bAVM rupture by the logistic regression (LR) algorithm and random forest (RF) algorithm. The area under the curve (AUC) was used to evaluate the predictive performances of those models.

**Results:**

The performances of the prediction models built by both algorithms were not ideal (AUCs: 0.7 or less). The AUCs from the models built by the LR algorithm with different sample sizes were better than those built by the RF algorithm (0.70 vs 0.68, *p* < 0.001). The standard deviations (SDs) of the AUCs from both prediction models with different sample sizes displayed wide ranges (max range > 0.1).

**Conclusions:**

Based on the current risk predictors, it may be difficult to build a stable and accurate prediction model for the hemorrhagic risk of bAVMs. Compared with sample size and algorithms, meaningful predictors are more important in establishing an accurate and stable prediction model.

## Background

Brain arteriovenous malformation (bAVM) is a cerebrovascular disease characterized by direct shunts between arteries and veins and abnormal vascular masses [1]. The main presenting clinical symptoms are hemorrhage and epilepsy. Because of the high mortality and disability associated with bAVMs rupture in many cases, particularly how to prevent and treat rupture, is always the focus of research. However, whether to intervene when bAVMs occur is still controversial [2–4]. Sometimes both the rupture rate of bAVMs in patients and the risk of endovascular or surgical treatment(when radiosurgery is not appropriate) are not low, it is important to assess the risk of rupture more cautiously before treatment.

The common method of developing a prediction model or a scoring system for disease risk is to build a mathematical model based on correlated clinical predictors. For binary category data, multivariate logistic regression (LR) is the conventional algorithm [5, 6]. With the development of computational algorithms, different machine learning methods have been introduced into this field [7]. Of them, random forest (RF) is considered to be a promising method. Previous studies on predicting the risk of diseases have reported many successful cases in which RF was applied [8, 9].

In this study, we collected the clinical data of 353 patients with bAVMs and built prediction models by the LR algorithm and RF algorithm based on multiple random samplings and different training sample sizes, and areas under the curve (AUCs) were used to assess the performances of the models. The purpose of our study is to test and compare the stability and performances of prediction models built by both algorithms and to investigate the deficiencies in these prediction models.

## Methods

### Case selection and data collection

All patients with bAVMs confirmed by digital subtraction angiography (DSA) from January 2013 to December 2019 were enrolled in our study. Patients with the following conditions were excluded: 1) a combination with brain injury or brain tumors; and 2) incomplete clinical data. Variables that were reported to be correlated with bAVM rupture in previous studies were collected [1, 6, 10]. General variables including age and sex were collected, and morphological variables pertaining to the bAVMs were separately measured on DSA images by 2 neurosurgeons (Wengui Tao and Laochao Yan), including the location, size, associated aneurysm, draining type, and number of draining veins. Other variables, including rupture information, were recorded.

All procedures in this retrospective study that involved human participants were approved by the ethical committee of Xiangya hospital and performed in accordance with the institutional ethical standards, the 1964 Helsinki declaration and its later amendments, or comparable ethical standards.

### Building prediction models by the LR algorithm and RF algorithm based on multiple repeated samplings and different sample sizes

RStudio (version 1.1.383; RStudio Inc.) was used to build the prediction models. Variables including sex, location, correlated aneurysm, draining type, and rupture were set as factor (categorical) variables, and variables including age, size, and the number of draining veins were set as numeric (continuous) variables. Rupture was set as the dependent (response) variable, and the other 7 variables were set as independent (explanatory) variables. In the LR algorithm, the independent variables were filtered by the step method, and significant variables were finally used for the predicting formula. In the RF algorithm, default values were set for the "ntree" and "mtry" parameters (500 and 3).

According to the 10 events per variable (EPV) rule [11–13], we sampled different sizes of training datasets from all 353 cases each time, and the remaining cases were defined as test datasets. The sample sizes of the training datasets were 140, 175, 210, 245 and 280, and the corresponding test datasets were 213, 178, 143, 108 and 73. For each pair of datasets, the number of random sampling times was 1, 10, 50, 100, 300, 600, 1200 and 2100.

### Calculating AUCs to assess the performances of prediction models

AUCs were used to assess the performances of the prediction models. The mean ± standard deviations (SD) was used to depict the AUCs.

After the source code was confirmed, multiple samplings, building the models, predictions, calculating the AUCs and plotting were fulfilled by a computer.

### Statistical analysis

Paired sample T-tests were used to compare the AUCs that resulted from the different prediction models built by the LR and RF algorithms. A *p *value < 0.05 was considered to be statistically significant.

## Results

### Demographics

The clinical data of 353 patients with ruptured and unruptured bAVMs are summarized in Table 1. Of all patients, 220 were male, and 133 were female, with a mean age of 32.82 ± 15.77 years. A total of 264 (74.8%) bAVMs were located in the cerebral lobes (superficial), 40 (11.3%) in the corpus callosum, basal ganglia or lateral ventricle (deep), and 49 (13.9%) in the cerebellum or brain stem (infratentorial). Ten (5.4%) patients had aneurysms related to bAVMs. The mean size of the bAVM nidus was 3.71 ± 2.15 cm. Seventy-four (21.0%) patients only had deep draining veins. A total of 198 (43.9%) patients only had single draining veins. BAVMs in 228 patients were confirmed to be ruptured and 125 unruptured.

**Table 1:** Summary of the clinical data

	Overall (n = 353)	Unruptured (n = 125)	Ruptured (n = 228)	*p *value
Age [mean (SD)]	32.82 (15.77)	35.14 (14.10)	31.55 (16.50)	0.041*
Sex = male (%)	220 (62.3)	84 (67.2)	136 (59.6)	0.199
Location (%)				< 0.001*
Superficial	264 (74.8)	115 (92.0)	149 (65.4)	
Deep	40 (11.3)	4 (3.2)	36 (15.8)	
Infratentorial	49 (13.9)	6 (4.8)	43 (18.9)	
Associated aneurysm (%)	19 (5.4)	1 (0.8)	18 (7.9)	0.01*
Size [mean (SD)]	3.71 (2.15)	4.39 (2.02)	3.33 (2.13)	< 0.001*
Draining type = deep (%)	74 (21.0)	20 (16.0)	54 (23.7)	0.119
Draining veins = multiple (%)	155 (43.9)	65 (52.0)	90 (39.5)	0.031*

*p value < 0.05: statistically significant

### Univariate analysis

Univariate analysis showed that age, location, associated aneurysm, size and the number of draining veins were significantly different between patients with unruptured and ruptured bAVMs. All these variables were used in LR and RF analyses.

### Performances of the prediction models

**Fig. 1:**
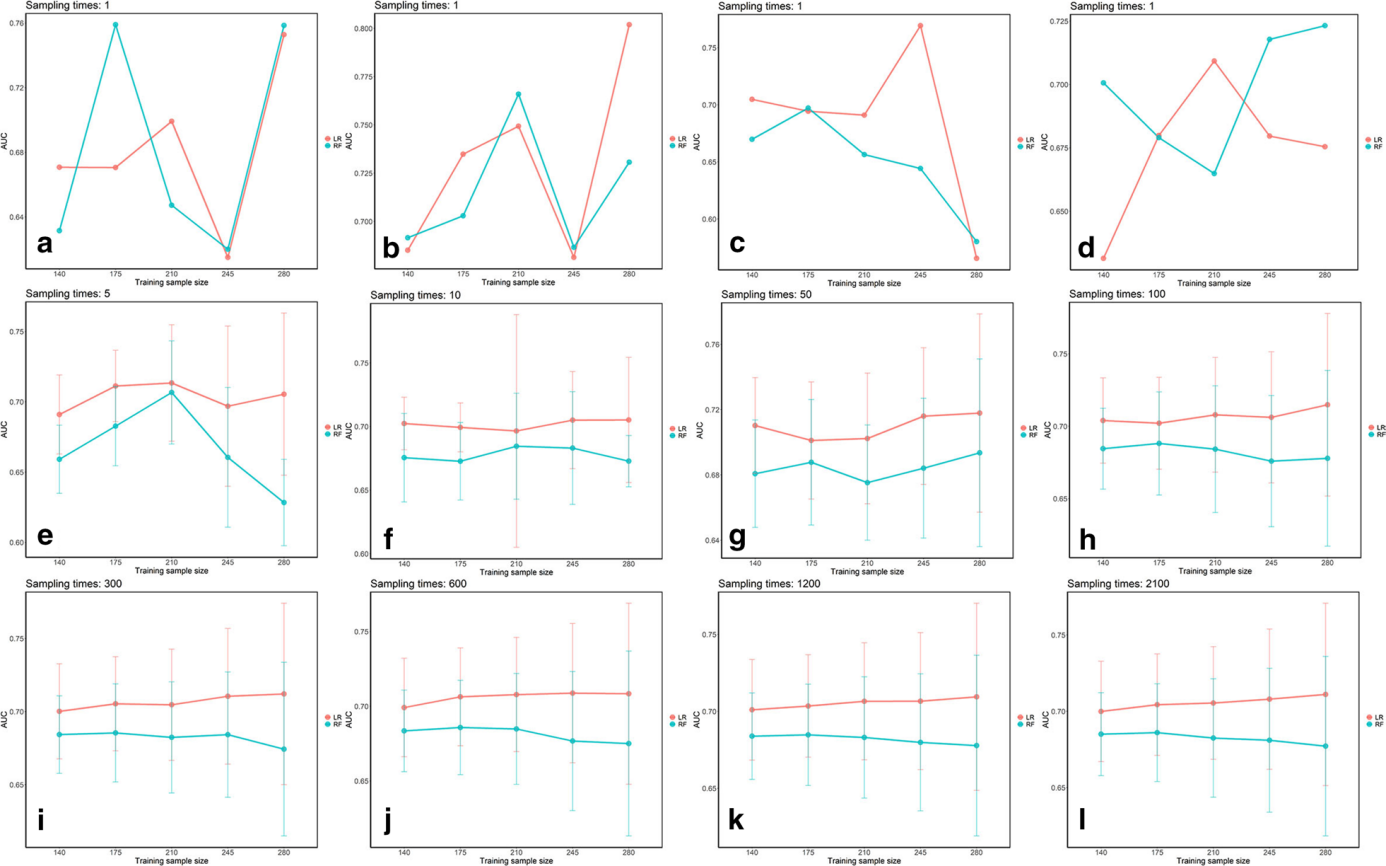
AUCs for the mean ± SD with the training sample size and changes in the sampling times. **a**–**d** The instability of the prediction models built by the LR algorithm (red line) and RF algorithm (blue line) based on different single sampling times and sample sizes.** a-l** show that the prediction models built by the LR algorithm were better than those built by the RF algorithm. AUCs above 100 samplings showed that the performances of the prediction models built using the LR algorithm could be slightly improved as the training sample size increased, but the RF algorithm demonstrated the opposite performance. SDs of the AUCs from the prediction models built by both algorithms with different sample sizes displayed wide ranges.** a-l** separately represent the sampling times: 1, 1, 1, 1, 5, 10, 50, 100, 300, 600, 1200, and 2100 (related data are shown in Table 2). *AUC* area under the curve, *LR* logistic regression, *RF* random forest, *SD* standard deviations

All the AUCs showed that the performances of the prediction models built by the LR algorithm were better than those built by the RF algorithm (*p* < 0.001), see Fig. 1 and Table 2. The AUC results showed that while the training sample size increased in the LR algorithm, the AUCs were slightly improved from 0.70 to 0.71 (> 100 sampling times). However, in the RF algorithm, the AUCs decreased. The standard deviations (SDs) of the AUCs showed a maximum fluctuation range > 0.1 in different samplings, and different single samplings also reflected unstable performances of the prediction models (see the first row of Fig. 1).

**Table 2:** AUCs of prediction models based on different training sample sizes and multiple sampling times

Algorithm	LR					RF					*p* value
Training sample size/test sample size	140/213	175/178	210/143	245/108	280/73	140/213	175/178	210/143	245/108	280/73	
Sampling times_1-1 [mean (SD)]	0.67 (NA)	0.67 (NA)	0.70 (NA)	0.61 (NA)	0.75 (NA)	0.63 (NA)	0.76 (NA)	0.65 (NA)	0.62 (NA)	0.76 (NA)	< 0.001
Sampling times_1-2 [mean (SD)]	0.69 (NA)	0.73 (NA)	0.75 (NA)	0.68 (NA)	0.80 (NA)	0.69 (NA)	0.70 (NA)	0.77 (NA)	0.69 (NA)	0.73 (NA)	
Sampling times_1-3 [mean (SD)]	0.70 (NA)	0.69 (NA)	0.69 (NA)	0.77 (NA)	0.57 (NA)	0.67 (NA)	0.70 (NA)	0.66 (NA)	0.64 (NA)	0.58 (NA)	
Sampling times_1-4 [mean (SD)]	0.63 (NA)	0.68 (NA)	0.71 (NA)	0.68 (NA)	0.68 (NA)	0.70 (NA)	0.68 (NA)	0.66 (NA)	0.72 (NA)	0.72 (NA)	
Sampling times_5 [mean (SD)]	0.69 (0.028)	0.71 (0.025)	0.71 (0.041)	0.70 (0.057)	0.71 (0.058)	0.66 (0.024)	0.68 (0.028)	0.71 (0.037)	0.66 (0.050)	0.63 (0.031)	
Sampling times_10 [mean (SD)]	0.70 (0.021)	0.70 (0.019)	0.70 (0.091)	0.71 (0.038)	0.71 (0.049)	0.68 (0.035)	0.67 (0.030)	0.68 (0.042)	0.68 (0.044)	0.67 (0.020)	
Sampling times_50 [mean (SD)]	0.71 (0.029)	0.70 (0.036)	0.70 (0.040)	0.72 (0.042)	0.72 (0.061)	0.68 (0.033)	0.69 (0.038)	0.68 (0.035)	0.68 (0.043)	0.69 (0.058)	
Sampling times_100 [mean (SD)]	0.70 (0.029)	0.70 (0.032)	0.71 (0.040)	0.71 (0.045)	0.71 (0.063)	0.68 (0.028)	0.69 (0.036)	0.68 (0.044)	0.68 (0.045)	0.68 (0.061)	
Sampling times_300 [mean (SD)]	0.70 (0.033)	0.71 (0.032)	0.70 (0.038)	0.71 (0.046)	0.71 (0.062)	0.68 (0.027)	0.69 (0.034)	0.68 (0.038)	0.68 (0.043)	0.67 (0.059)	
Sampling times_600 [mean (SD)]	0.70 (0.033)	0.71 (0.033)	0.71 (0.038)	0.71 (0.047)	0.71 (0.061)	0.68 (0.027)	0.69 (0.032)	0.68 (0.037)	0.68 (0.047)	0.68 (0.062)	
Sampling times_1200 [mean (SD)]	0.70 (0.033)	0.70 (0.033)	0.71 (0.038)	0.71 (0.044)	0.71 (0.061)	0.68 (0.028)	0.68 (0.033)	0.68 (0.039)	0.68 (0.044)	0.68 (0.059)	
Sampling times_2100 [mean (SD)]	0.70 (0.033)	0.70 (0.033)	0.71 (0.037)	0.71 (0.046)	0.71 (0.060)	0.68 (0.027)	0.69 (0.032)	0.68 (0.039)	0.68 (0.047)	0.68 (0.059)	

This table corresponds to Fig. 1

*AUC* area under the curve, *LR* logistic regression, *RF* random forest, *SD* standard deviations

## Discussion

BAVMs represent an intracranial hemorrhagic disease. The annual rupture rate of bAVMs reported in various literature is different [14–18]. For each patient and lesion, the risk of rupture should be assessed separately. Of patients who survive after the initial hemorrhage, approximately 20% die, and one-third remain moderately disabled after 3 months [1]. For patients with unruptured bAVMs, the psychological impacts associated with the long-term fear of hemorrhage should not be underestimated [19]. Additionally, it is necessary to compare the risk of bAVMs rupture with that of treatment. All these showed that predicting the hemorrhagic risk was important for unruptured bAVMs. Some studies proposed predictors for hemorrhagic risk, such as female sex, deep location, deep draining veins, single draining veins, and associated aneurysm [20–23]. Depending on these predictors, some authors tried to develop prediction models or scoring systems for the hemorrhagic risk of bAVM [6]. A successful prediction model or a scoring system would help clinical workers find suitable and low-risk management options for patients.

For binary categorical clinical data, the LR algorithm is the conventional method for building prediction models [5]. In recent years, machine learning algorithms have been introduced in this field. The highly accurate results and simplified procedures that resulted from the introduction of these methods are impressive. Of these machine learning algorithms, the RF algorithm is considered most promising because of its better performance, especially for big data [24].

The common method for building a prediction model is to obtain a training dataset from the whole data by date sequence or randomly and then to build a model in the form of a predicting formula (LR) or a predicting procedure hidden in black boxes (machine learning). The remaining data are defined as the test dataset and used to test the model. The AUC is usually used to evaluate predicting performances. The training sample size of the training dataset should meet the basic request of the 10 events per variable (EPV) rule [11–13].

In this study, our original purpose was to try to build prediction models for predicting the risk of bAVM rupture by the LR algorithm and RF algorithm and to compare the performances of those models. However, the results were not as expected, and the models displayed instability and uncertainty. When we performed multiple random samplings for the training dataset, the coefficients of the prediction formula from the LR algorithm varied, and the AUC also displayed different values, as did the RF algorithm. To explore this problem further, we increased the number of sampling times, changed the ratio of the training sample size to the test sample size, and even changed the number of independent variables; additionally, we observed the change in AUCs and tried to identify rules. Although the AUCs were widely dispersed with varying sample sizes and random sampling times, they still displayed certain patterns. Being familiar with these patterns can help us understand the possible uncertainty and instability of prediction models, help us build optimal prediction models, and avoid pitfalls.

The independent variables (explanatory variables) used in this study have been accepted by most researchers and are considered to be risk factors for bAVM rupture [1, 6, 10], but their performances in predicting hemorrhage were not ideal in this study. Their deficiencies did not radically change regardless of the algorithms we used or the increased sampling times or different training sample sizes. We believed that obtaining an ideal prediction model for predicting bAVM rupture might depend on the identification of new, more valuable predictors.

According to statistics, it is generally considered that if we try to obtain an effective result in regression analysis, the sample size should meet the 10 EPV rule. Our study showed that if the training sample size for the LR algorithm was increased on the basis of the 10 EPV rule, the predicting performance would only be improved slightly. This result indirectly proved the 10 EPV rule. Although the RF algorithm has shown advantages in many studies, in this study, its performance was not better than that of the LR algorithm. This result suggested that if there were not some significant independent variables, it would also be difficult for the RF algorithm to display its power.

In most previous studies on prediction models, the training dataset was almost always based on a single random sampling or date order; in fact, the number of sampling times was not specified in the statistics [5, 6]. However, in our study, the SDs reflected the instability that resulted from different samplings.

This study was based on clinical data from 353 patients with bAVMs; limitations in the sample size may affect the conclusions, and data were collected from a single center. The reliability and generality of the conclusions should be verified in a multicenter study.

## Conclusions

Both the prediction model by LR algorithm or RF algorithm based on the current risk predictors are not ideal. Compared with sample size and algorithms, meaningful predictors are more important in establishing an accurate and stable predictive model.

## Data Availability

The data that support the findings of this study are available from the corresponding author upon reasonable request.

## Abbreviations

bAVM: Brain arteriovenous malformation
LR: Logistic regression
RF: Random forest
AUC: Area under the curve
SD: Standard deviation
ARUBA: A Randomized Trial of Unruptured Brain Arteriovenous Malformation
EPV: Events per variable
DSA: Digital subtraction angiography
